# Nutritional Composition, Mineral Profile, Bioactive Phytochemicals, and Antioxidant Activities of Selected Edible Weeds: A Comprehensive Analysis

**DOI:** 10.1002/fsn3.70635

**Published:** 2025-07-15

**Authors:** Md. Mostafa Kamal, Somiya Haque, Tanim Kazi Suvo, Prity Akter, Md. Suman Mia, Md S. M. Sifat Shah, Md. Nahidul Islam, Md. Golam Ferdous Chowdhury

**Affiliations:** ^1^ Department of Food Engineering Gopalganj Science and Technology University Gopalganj Bangladesh; ^2^ Department of Food Engineering Gazipur Agricultural University Gazipur Bangladesh; ^3^ Institute of Food Safety and Processing Gazipur Agricultural University Gazipur Bangladesh; ^4^ Postharvest Technology Division Bangladesh Agricultural Research Institute Gazipur Bangladesh

**Keywords:** bioactive components, chlorophyll, functional foods, heavy metals, nutrition

## Abstract

Edible weeds are increasingly recognized as rich sources of bioactive phytochemicals and antioxidants, contributing to health benefits and playing important roles in traditional medicine and modern nutrition. This study evaluated the chemical composition, mineral content, and antioxidant properties of powdered forms of seven edible weeds from Bangladesh. Additionally, the health risks associated with trace elements in these weeds were assessed. Significant variations (*p* < 0.05) in the chemical composition were observed, with moisture ranging from 7.02% to 13.64%, ash from 10.14% to 19.30%, protein from 7.05% to 13.95%, fat from 1.53% to 6.51%, carbohydrate from 58.81% to 76.73%, and energy values from 378.91 to 486.98 kCal/100 g. Phytochemical screening revealed notable amounts of ascorbic acid (21.53 to 156.66 mg/100 g), total chlorophyll (7.35 to 7.68 mg/100 g), total carotenoids (46.24 to 1973.72 mg/100 g), total phenolics (685.73 to 3046.67 mg GAE/100 g), and flavonoids (154.28 to 1973.72 mg CE/100 g). Antioxidant activities were strong, with DPPH radical scavenging activity values ranging from 58.15% to 72.77% and reducing power assay results from 6328.00 to 12298.23 mg TE/100 g. Although the weed powders contained substantial minerals, certain species showed high trace element accumulation, posing potential health risks. The findings highlight edible weeds as functional foods with significant bioactive substances and potent antioxidant properties. However, monitoring safety to mitigate health risks from trace elements is crucial. This study serves as a basis for developing dietary guidelines and exploring the commercial potential of edible weeds in food and pharmaceutical industries.

## Graphical abstract

Edible weeds from Bangladesh have been evaluated and found to contain significant amounts of nutrients, bioactive phytochemicals, and antioxidants, supporting their potential as valuable alternative sources of functional foods. However, some species exhibit elevated levels of trace metals, posing health risks. Overall, these weeds offer valuable nutraceutical prospects but require safety assessments to ensure contaminated element levels are within permissible limits for safe consumption.

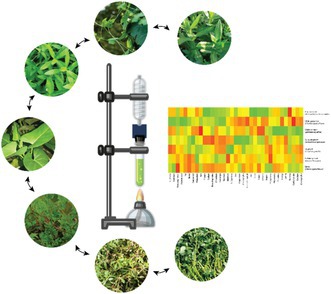

## 1 Introduction

Reactive oxygen species (ROS), including hydroxyl radicals, hydrogen peroxide, singlet oxygen, and superoxide, are by‐products of normal cellular metabolism that play crucial roles in cell signaling and homeostasis (Mittal et al. 2014). Excessive ROS production—due to environmental stressors, pollution, and improper food processing—can lead to oxidative damage, contributing to the pathogenesis of various chronic diseases including cancer, cardiovascular disorders, neurodegenerative diseases, and aging (Tanase et al. 2019; Zhang et al. 2018). Therefore, identifying natural, plant‐based sources with potent antioxidant properties is a key strategy for disease prevention and health promotion.

In recent years, there has been a growing interest within the scientific community and the food industry to explore edible weeds as sustainable sources of bioactive phytochemicals and nutrients. This trend is driven by concerns over the adverse effects of synthetic medications, which are linked to the resistance of pathogens (Karasawa and Mohan 2018). Plants and their components serve as a vital source of medicines and are effective in treating numerous illnesses, primarily due to their content of phytochemicals such as polyphenols, flavonoids, carotenoids, chlorophyll, and anthocyanins. These compounds, along with antioxidants, vitamins, minerals, and dietary fibers, play essential roles in mitigating health complications.

Weeds, typically growing unwanted alongside cultivated crops, are often regarded negatively due to their invasive nature affecting commercial crops (Sams et al. 2023). However, several edible weeds, including Water Spinach ( *Ipomoea aquatica* ), Gima (*Glinus oppositifolius*), Alligator Weed ( *Alternanthera philoxeroides* ), Buffalo Spinach (*Enydra fluctuans*), Ivy Gourd ( *Coccinia grandis* ), White Goosefoot ( *Chenopodium album* ), and Spiny Amaranth ( *Amaranthus spinosus* ), can serve as valuable culinary ingredients and contribute to household food supplies, especially in tropical regions where they are frequently consumed as staple foods (Stark et al. 2019).

These weeds are consumed not only for their nutritional benefits, but also used as traditional medicines. They contain diverse phytochemical compounds with medicinal properties (Freitas et al. 2021; Sarker and Oba 2019). Additionally, these weeds are rich sources of nutrients such as vitamins, minerals, fiber, and secondary metabolites, offering functional benefits when included in food formulations. Consequently, research into the extraction and identification of natural compounds and antioxidants from these edible weeds holds significant potential for applications in the food, cosmetic, and pharmaceutical industries.

However, the presence of hazardous metals in edible weeds may pose potential health risks. Elements like chromium, nickel, arsenic, mercury, aluminum, cadmium, and lead are toxic with extended biological half‐lives and non‐biodegradability, thereby posing serious health risks. Contaminated edible weeds may cause health risks through slow poisoning, affecting organs like the kidney, bone, liver, and healthy cells when consumed in high concentrations over the years (Rahman and Islam 2019; Sultana et al. 2017; van der Walt et al. 2008). Therefore, determining the concentrations of these hazardous elements in edible weeds is crucial to ensure consumer safety.

Existing studies have demonstrated the rich phytochemical profiles of various edible weeds, including *Amaranthus* spp., *Chenopodium album* , and *Ipomoea aquatica* , revealing their high contents of antioxidants, phenolics, flavonoids, and vitamins, which confer significant health benefits (Sarker and Oba 2019; Sarker et al. 2024; Singh et al. 2023); (Saikia et al. 2023). For instance, recent research shows that *Amaranthus* species possess notable antioxidant capacities and therapeutic potentials, while *C. album* exhibits antifungal, anti‐inflammatory, and antidiabetic properties (Dey et al. 2025; Meenatchi et al. 2017; Sarker et al. 2024). Additionally, studies from different regions have highlighted the occurrence of heavy metals in leafy vegetables, underscoring the importance of monitoring toxic element levels to safeguard public health.

Although existing studies have documented the nutritional and phytochemical properties of specific weedy species (Gqaza et al. 2013; Guerrero and Torija Isasa 1997), there is a lack of integrated research focusing on Bangladeshi edible weeds, particularly assessing both beneficial bioactive compounds and potential toxic elements. This study aims to fill this critical gap by providing a comprehensive evaluation of the nutritional, mineral, and phytochemical profiles of seven locally available edible weeds, along with the assessment of antioxidant activity and trace metal safety.

## 2 Materials and Methods

### 2.1 Raw Materials

Edible leafy part of total of seven weeds— *Ipomoea aquatica* , *Glinus oppositifolius*, *Alternanthera philoxeroides* , *Enydra fluctuans*, *Coccinia grandis* , *Chenopodium album* , and *Amaranthus spinosus* —was collected from various locations (latitude 22° 57′ 58″ N and longitude 89°48′44″ E) in Gopalganj, Bangladesh. The weeds were conventionally grown and no additional fertilization or irrigation was applied. The selection of the location was based on their prevalence and traditional consumption status in local communities. A total of 200 individual plants per species were sampled across different microenvironments to account for intra‐species variability. Following the collection, any adhering dirt or debris was carefully removed, and the samples were subsequently washed with tap water and allowed to dry in the shade with forced air (average atmospheric temperature 27.93°C and RH 69.8%). Drying was carried out until the samples reached a stable weight. Once dried, the samples were pulverized into fine powder using a laboratory grinder (Jaipan CM/L‐7360065, Japan). The resulting powder was then sieved, packaged in high‐density polyethylene bags, and stored at 4°C in the refrigerator until analysis for proximate compositions, mineral profiles, and the extraction and analysis of bioactive phytochemicals. Table 1 provides an overview of wild edible weed samples.

**TABLE 1:** Experimental wild edible weed samples in different phases.

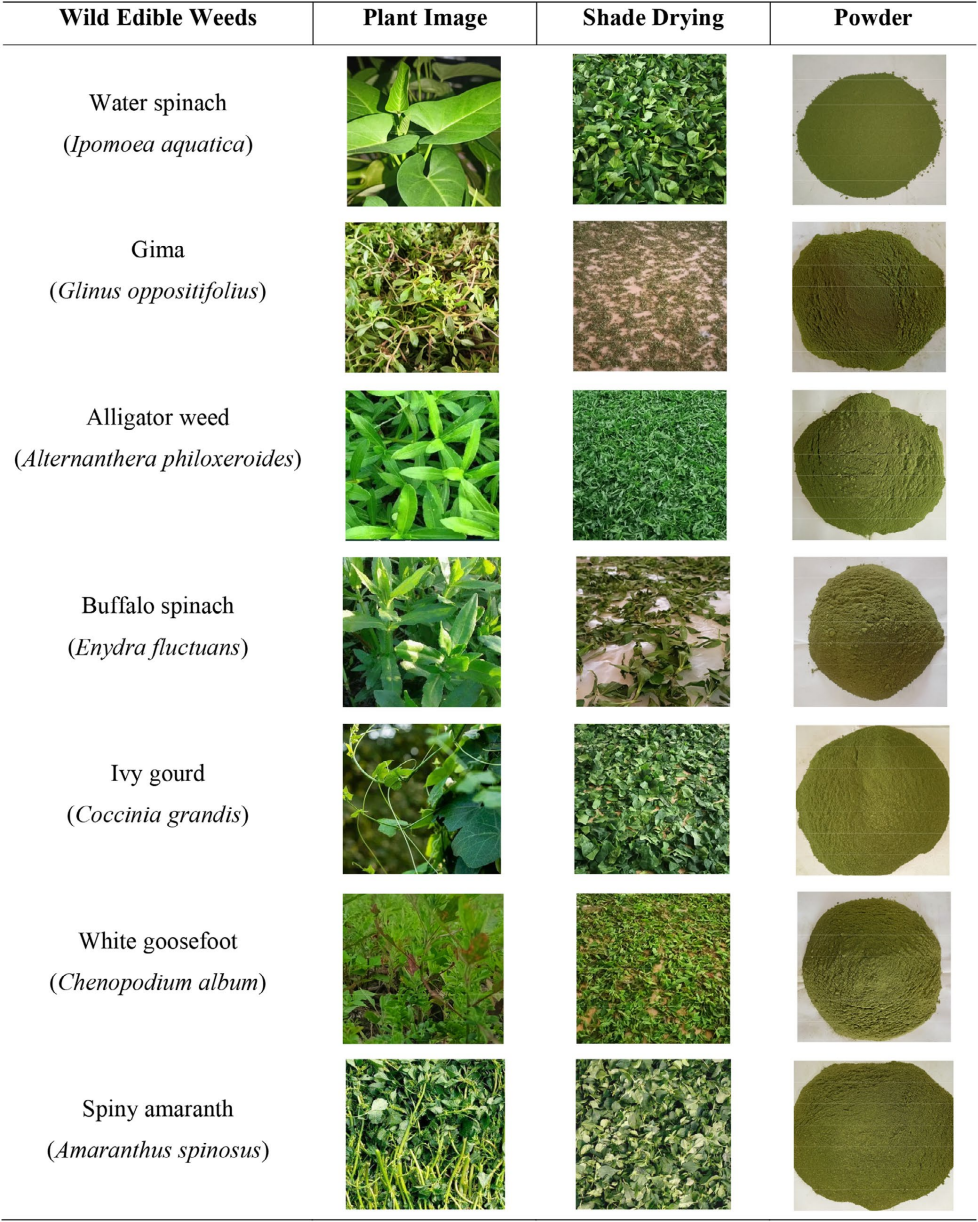

### 2.2 Determination of Chemical Composition

Instrumental calibration and method validation were performed prior to sample analysis, following established protocols. Calibration curves were freshly prepared for every batch, and control samples were analyzed periodically within each analytical run to verify instrument stability. The measurements of moisture, protein, ash, and fat content were conducted using established standard protocols and expressed as percentages on a dry basis (db) (Rahman et al. 2024). Nitrogen content was determined using the Kjeldahl method, and protein content was calculated by multiplying nitrogen content by 6.25 (Ali et al. 2022). The carbohydrate content was determined using the difference method.

Furthermore, the energy content of the samples was calculated utilizing a bomb calorimeter (Parr 6100 Calorimeter, USA). For this process, 1 g of weed powder was placed into the bomb head, which was then inserted into the bomb cylinder. The O_2_ filling line was connected, and two liters of water were added to the bucket. Subsequently, the bomb cylinder was securely placed inside the bomb calorimeter, and the cap was closed tightly. Afterward, the calorimeter was activated, allowing the energy value to be recorded directly from the screen and expressed as kCal per 100 g of the sample (Hasan et al. 2022).

### 2.3 Mineral Profiling

For mineral analysis, the wild edible weed powder was first converted into wet ash by digesting with a solution of HNO_3_ and HClO_4_ in a 5:1 ratio (at 120°C for 1 h then at 180°C for another 1 h). After that, the solution was cooled and filtered using Whatman No. 42 filter paper. The filtrate was then diluted to a volume of 30 mL to serve as a stock solution for further analysis.

Calcium (Ca), magnesium (Mg), sodium (Na), copper (Cu), iron (Fe), zinc (Zn), lead (Pb), cadmium (Cd), nickel (Ni), chromium (Cr), and arsenic (As) were determined using an atomic absorption spectrophotometer (AA‐7000S, Shimadzu, Tokyo, Japan). Meanwhile, potassium (K) was quantified using flame photometry (SpectrAA, 55B, Varian, USA). In addition, phosphorus (P) and sulfur (S) contents were measured through the spectrophotometric technique (Specord 205, Analytik Jena, Germany) (S. Ahmed et al. 2023; Kabir et al. 2024).

### 2.4 Measurement of Color Parameters

The color of weed powder samples was determined using a chroma meter (CR410, Konica Minolta Inc., Japan). The color characteristics of the edible weed powders were identified as L* (lightness), a* (red/green), b* (yellow/blue), hue angle (H) and chroma (C) values. Triplicate measurements were taken for each sample. The following equation was used to calculate the Browning index based on the color coordinates (L*, a*, and b*) (Islam et al. 2014).

$$\text{Browning Index}=\left(\frac{X-0.31}{0.17}\right)\times 100$$

$$\text{Where}\;X=\frac{\left(a^{*}+1.75L^{*}\right)}{5.645L^{*}+a^{*}-3.012b^{*}}$$

### 2.5 Determination of Bioactive Compounds

To extract bioactive compounds, sample solutions were heated using a water bath (Vision Scientific Co. Ltd., Korea) equipped with a thermostat for precise temperature control. Five grams of the powder sample were placed in a conical flask and mixed with 100 mL of deionized water, maintaining a powder‐to‐liquid ratio of 1:20. This heating process was carried out for 30 min at 70°C with continuous shaking at 100 rpm.

After that, the resulting slurry was vacuum‐filtered using Whatman No. 1 filter paper, followed by centrifugation (himac CT6E, Hitachi Koki Co. Ltd., Japan) for 10 min at 4000 *g*. The clear supernatant obtained from this process was collected in a falcon tube and stored at‐18°C for subsequent phenolic and flavonoid analysis (Kumer et al. 2024).

#### 2.5.1 Determination of Total Phenolic Content

The Folin–Ciocalteu reagent method was employed to determine the total phenolic content of the sample extracts, following the procedure outlined by Mahomud et al. (2024). Briefly, 0.5 mL of 10% (v/v) Folin–Ciocalteu reagent was added to 0.5 mL of the sample extract in a centrifuge tube and thoroughly mixed. To neutralize the mixture, 8 mL of distilled water and 1 mL of 7.5% Na_2_CO_3_ were added. After vortexing, the tubes were centrifuged at 4000 × *g* for 10 min and subsequently left in the dark at room temperature for 35 min.

The absorbance of the supernatant was measured at 725 nm using a UV/Vis spectrophotometer (Jasco, V‐630, Japan) compared to a blank. A calibration curve for determining the total phenolic content was prepared using standard gallic acid, and the final results were expressed as milligrams of gallic acid equivalent (GAE) per 100 g (mg GAE/100 g) of the sample.

#### 2.5.2 Determination of Total Flavonoid Content

The total flavonoid content of the sample extracts was determined using a modified version of the previously published protocol by Zhou et al. (2021). Specifically, 1 mL of the sample extract, 4 mL of deionized water, and 0.3 mL of 5% NaNO_2_ were combined in a centrifuge tube and thoroughly mixed. To enhance the reaction, the mixture was allowed to rest for 5 min. Following this, 0.3 mL of 10% AlCl_3_ was added, and the mixture was allowed to settle for an additional minute.

After thorough mixing of the solution, 2.4 mL of distilled water and 2 mL of 1 M NaOH were added. The mixture was then centrifuged for 5 min at 4000 × *g*, and the supernatant was separated using a 10 mL plastic syringe. The supernatant was stored in the dark at room temperature for 15 min before the absorbance was measured against a blank at a wavelength of 510 nm using a UV/Vis spectrophotometer (Jasco, V‐630, Japan). A standard curve based on catechin was prepared to calculate the total flavonoid content, and the results were expressed as milligrams of catechin equivalents (mg CE/100 g) per 100 g of sample.

#### 2.5.3 Estimation of Ascorbic Acid Content

To assess the content of ascorbic acid, a 2,6‐dichlorophenol indophenol titration method, with some modifications, was applied (Cao et al. 2020). In brief, 5 mL of a 20% w/v metaphosphoric acid solution was mixed with 2 g of the powder sample, followed by filtration. An aliquot of 1 mL from the filtered solution was then combined with 10 mL of distilled water in a beaker.

Subsequently, 2 mL of this mixture was placed into a separate beaker, where two drops of phenolphthalein indicator were added. The resulting solution was titrated against the 2,6‐dichlorophenol indophenol dye until the appearance of a faint pink color indicated the endpoint of the titration. Ascorbic acid content was expressed as milligrams of ascorbic acid per kilogram of fresh sample.

$$\text{Ascorbic acid}\;\left(\mathrm{mg}/100g\right)=\frac{T\times \mathrm{DF}\times V_{1}}{W\times V_{2}}\times 100$$

where, T‐Titre volume, DF‐Dye Factor [Dye factor = 0.5/Titre], W‐Weight of the sample, V_1_‐Volume made up, and V_2_‐Aliquot taken.

#### 2.5.4 Determination of Total Carotenoid Content (TCC)

Total carotenoid content (TCC) was determined following the methodology outlined in the study of Roy et al. (2024) with slight modification. Briefly, a 50 mL centrifuge tube was filled with 20 mL of ethanol‐hexane solution (70:30) and 2 g of the powder. After thoroughly homogenizing the mixture, it was centrifuged at 4000 × *g* for 15 min. The upper hexane layer, or supernatant, was then collected in a separate centrifuge tube, while the remaining liquid was poured off.

This extraction process was repeated two additional times, and the resulting supernatants were combined. Subsequently, the combined supernatant was mixed with 20 mL of distilled water and subjected to thorough mixing before being frozen overnight. On the following day, the liquid hexane layer was collected and mixed properly.

Afterward, the absorbance of the supernatant was measured at 470 nm. The total carotenoid content was then calculated using the following formula and expressed as milligrams per 100 g (mg/100 g) of the sample.

$$\mathrm{TCC}\;\left(\mathrm{mg}/100g\right)=\frac{{\text{Absorbance}}_{470\;\mathrm{nm}}\times \text{Total volume of hexane layer}\times 10,000}{\varepsilon \left(A_{1\mathrm{cm}}^{1\%}\right)\times \text{Sample weight}}$$

where, $\varepsilon \left(A_{1\mathrm{cm}}^{1\%}\right)$ is the extinction coefficient of the combination of carotenoids in hexane (2500 dL/g.cm).

#### 2.5.5 Determination of *β‐Carotene* and Chlorophyll Content

The chlorophyll (chlorophyll a, chlorophyll b and total chlorophyll) and *β‐carotene* content of the sample were measured using the methodology described by Cao et al. (2023). Specifically, 5 g of the powder sample were placed in a 100 mL conical flask and mixed with 50 mL of acetone‐hexane solution (4:6). After thorough homogenization, the mixture was centrifuged (himac CT6E, Hitachi Koki Co. Ltd., Japan) for 10 min at 4000 × *g*.

The resulting supernatant was collected in a falcon tube, and its absorbance was measured at wavelengths of 663, 645, 505, and 453 nm using a UV/VIS spectrometer (Jasco, V‐630, Japan).

The chlorophyll “a”, chlorophyll “b”, and total chlorophyll content were estimated and presented as mg/100 g by using the following equations

$$\text{Chlorophyll}\;a\;\left(\mathrm{mg}/100\;g\right)=0.999A_{663}-0.0989A_{645}$$

$$\text{Chlorophyll}\;b\;\left(\mathrm{mg}/100\;g\right)=-0.328A_{663}+1.77A_{645}$$

$$\text{Total Chlorophyll}\;\left(\mathrm{mg}/100\;g\right)=\text{Chlorophyll}\;a+\text{Chlorophyll}\;b$$

The *β‐carotene* content was estimated using the following equation and expressed as mg/100 g.

$$\beta ‐\text{Carotene}\;\left(\mathrm{mg}/100\;g\right)=0.216A_{663}-0.304A_{505}+0.452A_{453}$$

where, A663, A645, A505, and A453 are the absorbance at 663, 645, 505, and 453 nm, respectively.

### 2.6 Evaluation of Antioxidant Activity

In this study, the antioxidant properties of seven edible weeds were assessed using the DPPH radical scavenging activity (DPPH‐RSA) and a reducing power assay, following the methodology outlined by Kumer et al. (2024), with slight modifications. Specifically, 0.1 mL of the extract was taken in a centrifuge tube, to which 1.9 mL of a 0.3 mM DPPH solution was added and thoroughly mixed. The mixture was then kept in the dark at room temperature for 30 min, after which the absorbance of the solution was recorded at 517 nm using a UV/Vis spectrophotometer (Jasco, V‐630, Japan). The following equation was used to calculate the DPPH‐RSA, which is represented as a percentage of inhibition:

$$\%\text{Inhibition}=\frac{{\text{Absorbance}}_{\text{Control}}-{\text{Absorbance}}_{\text{Sample}}}{{\text{Absorbance}}_{\text{Control}}}\times 100$$

Moreover, the reducing power of the sample extracts was assessed following the protocol previously reported by Canabady‐Rochelle et al. (2015). In a centrifuge tube, 2.5 mL of phosphate buffer solution (0.2 M, pH 6.6) was combined with 2.5 mL of potassium ferricyanide (1%, w/v) and 0.5 mL of the sample extract. This mixture was incubated at 50°C for 20 min, after which 2.5 mL of 10% trichloroacetic acid was added. The mixture was then centrifuged at 1750 × *g* for 10 min.

Subsequently, 2.5 mL of the supernatant was taken and mixed with 2.5 mL of deionized water and 0.5 mL of a 0.1% (w/v) FeCl_3_ solution. The absorbance of the reaction mixture was measured at 700 nm using a UV/Vis spectrophotometer (Jasco, V‐630, Japan). The reducing power was calculated in comparison to a Trolox standard curve, with results expressed as milligrams of Trolox equivalents per 100 g (mg TE/100 g) of the sample.

### 2.7 Statistical Analysis

The number of samples analyzed was determined based on standard guidelines for nutritional analysis, which suggest a minimum of three replicates per species to ensure statistical robustness and reproducibility. Statistical analyses were conducted using IBM SPSS software (version 22.0, SPSS Inc., Chicago, USA) on data acquired for the tested quality metrics. Results were presented as mean ± standard deviation (STD). One‐way analysis of variance was carried out. To statistically assess significant variation between means with a 95% certainty level, Duncan Multiple Range Test (DMRT) was performed. Principal component analysis and cluster analysis were carried out using R statistical software (version 3.3.4) (Islam 2022).

## 3 Results and Discussion

### 3.1 Chemical Composition of Edible Weed Powder

In this study, fresh edible weed plants were dried and subsequently converted into powder for analysis. The proximate composition, including parameters such as moisture, ash, protein, fat, carbohydrate, and energy content, is presented in Table 2. The moisture content of the edible weed powder ranged from 7.02% to 13.64% on a dry mass basis (Table 2), with the highest moisture content recorded in Ivy gourd and the lowest in Buffalo spinach. Notably, the moisture content of the edible weed sample powder remains within the acceptable limit of less than 14%, which helps to preserve storage storability (Sagar and Kumar 2010). Previous literature indicates that higher moisture content in leafy vegetables can lead to increased susceptibility to microbial degradation due to the enhanced activity of water‐soluble enzymes (Datta et al. 2019). However, it is essential to recognize that factors such as temperature, humidity, and the harvesting period of the species significantly influence moisture content.

**TABLE 2:** Chemical composition of wild edible weeds powder.

Sample	Moisture (g/100 g)	Ash (g/100 g)	Protein (g/100 g)	Fat (g/100 g)	Carbohydrate (g/100 g)	Energy (kCal/100 g)
Water spinach	11.94 ± 0.43ab	10.14 ± 0.08d	8.88 ± 0.04c	4.25 ± 0.01b	76.73 ± 0.08a	486.98 ± 1.04a
Gima	10.39 ± 0.11bc	18.26 ± 0.08ab	7.05 ± 0.20d	1.85 ± 0.01d	72.83 ± 0.28ab	405.77 ± 0.93ab
Alligator weed	11.59 ± 1.04ab	14.99 ± 0.03c	13.95 ± 0.27a	1.53 ± 0.02e	69.53 ± 0.30b	444.58 ± 3.32ab
Buffalo spinach	7.02 ± 1.00e	16.27 ± 0.08bc	8.44 ± 0.12c	1.96 ± 0.02d	73.33 ± 0.21ab	422.12 ± 1.88ab
Ivy gourd	13.64 ± 0.13a	19.20 ± 0.04a	10.17 ± 0.06b	4.10 ± 0.04b	66.53 ± 0.07c	417.34 ± 2.11ab
White goosefoot	9.77 ± 0.15c	19.30 ± 0.07a	10.38 ± 0.17b	6.51 ± 0.27a	58.81 ± 0.22d	378.91 ± 0.68c
Spiny amaranth	8.57 ± 0.05d	16.65 ± 0.13bc	5.87 ± 0.17e	3.76 ± 0.22bc	71.73 ± 0.25ab	408.80 ± 0.30bc

*Note:* Values are represented as the mean ± standard deviation of the mean. Means followed by different lowercase letters in the same column indicate significant differences at *p* < 0.05.

Ash content represents inorganic plant materials, which include oxides and salts with various anions (such as phosphates, sulfates, and chlorides) and cations (like sodium, potassium, calcium, magnesium, iron, and manganese). The concentration of ash reflects the mineral profile of a sample. In this study, the ash content of the edible weed powders ranged between 10.14% and 19.30% (dry mass basis), with the highest content found in sample White goosefoot and the lowest in Water spinach. These findings correlate with results reported for other commonly consumed edibles from Bangladesh, Arunachal Pradesh, and Meghalaya in India (Mohammed Abdus Satter Mohammed Abdus et al. 2016; Seal et al. 2013, 2022). Although food processing techniques typically have minimal effects on minerals or ash content, such variations may arise from ecological conditions, mineral uptake patterns, and/or the age of the weed samples studied.

Protein, a vital component of diet, forms the structural basis for both human and animal bodies. As presented in Table 2, there was a significant difference (*p* < 0.05) in protein content among the various powders, with values ranging from 5.87% to 13.95% (dry mass basis). Powder of Alligator weed exhibited the highest protein content, whereas powder of Spiny amaranth showed the lowest.

Furthermore, the fat content varied from 1.53% to 6.51% on a dry mass basis, with White goosefoot showing the highest fat content, followed by Water spinach and Ivy gourd; conversely, Alligator weed had the lowest fat content among the weed powder samples.

The range of total carbohydrates (on a solid mass basis) was found to vary between 58.81% and 76.73%, with variations largely attributed to compositional differences among the weed samples. The energy content of the wild edible weed powder was substantial, with total energy values recorded between 378.91 and 486.98 kCal/100 g. As indicated in Table 2, Water spinach had the highest calorific value, while White goosefoot showed the lowest; nonetheless, other samples also exhibited significant calorific values.

### 3.2 Mineral Profiling of Edible Weed Powder

Minerals are essential for various physiological functions in the human body, including enzyme activity, nerve responses, muscle contraction, and blood clotting (Gupta et al. 2019). Table 3 summarizes the mineral composition of wild edible weed powder. The analysis revealed calcium concentrations ranging from 2.68 to 4.88 g/100 g, magnesium from 0.92 to 2.12 g/100 g, potassium from 0.11 to 1.52 g/100 g, sodium from 0.86 to 2.00 g/100 g, phosphorus from 1.11 to 3.68 g/100 g, and sulfur from 0.34 to 0.77 g/100 g across various weed powders (Table 3).

**TABLE 3:** Mineral and heavy metal profiling of wild edible weeds powder.

Sample ID	Mineral concentration (g/100 g)					
	Ca	Mg	K	Na	P	S
Water spinach	6.44 ± 0.02a	1.16 ± 0.01c	0.14 ± 0.001d	1.26 ± 0.01c	3.32 ± 0.02a	0.61 ± 0.01a
Gima	4.88 ± 0.01b	1.68 ± 0.01b	0.11 ± 0.001d	0.99 ± 0.01 cd	3.68 ± 0.03a	0.69 ± 0.01a
Alligator weed	3.16 ± 0.01c	1.4 ± 0.01b	0.22 ± 0.001c	2.00 ± 0.01a	3.02 ± 0.03a	0.77 ± 0.01a
Buffalo spinach	3.08 ± 0.01c	1.24 ± 0.01c	1.52 ± 0.01a	1.26 ± 0.01c	1.11 ± 0.01c	0.48 ± 0.01b
Ivy gourd	2.84 ± 0.01d	1.20 ± 0.01c	1.29 ± 0.01a	1.43 ± 0.01b	1.39 ± 0.01c	0.42 ± 0.01b
White goosefoot	2.68 ± 0.01d	0.92 ± 0.01d	0.80 ± 0.01b	1.56 ± 0.01b	2.64 ± 0.02b	0.40 ± 0.01b
Spiny amaranth	4.12 ± 0.02b	2.12 ± 0.01a	0.85 ± 0.01b	0.86 ± 0.01d	3.16 ± 0.03a	0.40 ± 0.01b

Sample ID	Heavy metal (mg/kg)							
	Cu	Fe	Zn	Pb	Cd	Ni	Cr	As
Water spinach	1.00 ± 0.02f	348.00 ± 7.33d	34.00 ± 2.34f	1.16 ± 0.01c	81.00 ± 1.05b	0.02 ± 0.001b	0.001 ± 0.0001b	0.36 ± 0.001d
Gima	2.00 ± 0.09e	428.00 ± 6.29c	60.00 ± 2.78e	1.56 ± 0.01c	93.00 ± 0.98a	0.01 ± 0.001c	0.001 ± 0.0001b	0.79 ± 0.001c
Alligator weed	9.00 ± 0.12a	620.00 ± 7.89b	522.00 ± 4.55a	5.28 ± 0.07a	82.00 ± 0.87b	0.02 ± 0.001b	0.002 ± 0.0001a	2.42 ± 0.03a
Buffalo spinach	6.00 ± 0.11c	325.00 ± 4.55d	90.00 ± 3.87d	1.24 ± 0.01c	83.00 ± 1.02b	0.01 ± 0.001c	0.002 ± 0.0001a	0.21 ± 0.001e
Ivy gourd	5.00 ± 0.13d	412.00 ± 5.33c	264.00 ± 2.67c	2.12 ± 0.02b	79.00 ± 0.89c	0.03 ± 0.001a	0.001 ± 0.0001b	0.24 ± 0.001e
White goosefoot	5.00 ± 0.11d	297.00 ± 4.56d	93.00 ± 2.12d	0.56 ± 0.01d	95.00 ± 1.37a	0.03 ± 0.001a	0.002 ± 0.0001a	0.09 ± 0.001f
Spiny amaranth	7.00 ± 0.16b	807.00 ± 8.91a	335.00 ± 3.69b	1.75 ± 0.02bc	85.00 ± 1.03b	0.03 ± 0.001a	0.001 ± 0.0001b	0.94 ± 0.01b
MPL	10.00	450.00	50.00	0.30	0.20	2.70	2.30	0.10
	(FAO/WHO 2013)	(FAO/WHO 2007)	(FAO/WHO 2013)	(FAO/WHO 2013)	(FAO/WHO 2001)	(FAO/WHO 2013)	(FAO/WHO 2013)	(FAO/WHO 2013)

*Note:* Values are represented as the mean ± standard deviation of the mean. Means followed by different lowercase letters in the same column indicate significant differences at *p* < 0.05.

Abbreviation: MPL, maximum permissible limit.

All samples of weed powder contained substantial amounts of these minerals, with significant variability observed among them. These variabilities may arise from the specific uptake capacity of different weed samples (McLaughlin et al. 2011). Importantly, the levels of mineral concentrations remained within safe dietary exposure limits, indicating that they are not detrimental to health (Jolly et al. 2013).

However, all weed powder samples also contained trace amounts of heavy metals. Specifically, copper concentrations ranged from 1.00 to 7.00 mg/kg, iron from 297.00 to 807.00 mg/kg, zinc from 34.00 to 522.00 mg/kg, lead varied between 0.56 and 5.28 mg/kg, cadmium ranged between 79.00 and 95.00 mg/kg, nickel ranged from 0.01 to 0.03 mg/kg, chromium from 0.001 to 0.002 mg/kg, and arsenic from 0.09 to 2.42 mg/kg (Table 3).

Given that many of the analyzed heavy metals exceed safe levels, consumers of edible weed powder may have valid concerns regarding potential health risks. Previous studies indicate that primary sources of heavy metal accumulation in food items include the excessive use of pesticides in agriculture, along with air, water, and soil pollution, as well as industrial discharges (Rahman et al. 2020; Sultana et al. 2017, 2022).

Future study should focus on long‐term monitoring of heavy metal accumulation in edible weeds across different regions, and the effectiveness of various preparation methods in reducing toxic elements. Additionally, clinical and in vivo studies are necessary to confirm health benefits and safety.

### 3.3 Evaluation of Color Attributes

Table 4 outlines the color attributes and browning index of wild edible weed powder. Color is a critical quality parameter often evaluated for dried products, significantly influencing consumer purchasing decisions. The lightness (L*) of the edible weed powder ranged from 43.10 to 54.36, with White goosefoot exhibiting the highest L* value. It is noteworthy that the brightness of edible weed powder showed significant variation (*p* < 0.05), except for the powders produced from Gima, Buffalo spinach, and Spiny amaranth. Conversely, samples Water spinach, Alligator weed, and Ivy gourd displayed the lowest lightness values, indicating a darker hue of the weed powder.

**TABLE 4:** Color attributes of wild edible weed powder.

Sample ID	L*	a*	b*	Hue angle	Chroma	Browning index (%)
Water spinach	43.10 ± 0.29bc	−5.74 ± 0.06b	25.32 ± 0.25bc	25.96 ± 0.26c	102.75 ± 0.04bc	71.81 ± 0.34b
Gima	49.50 ± 0.42ab	−5.72 ± 0.03b	22.16 ± 0.09c	22.88 ± 0.09d	104.46 ± 0.06ab	47.60 ± 0.37e
Alligator weed	44.84 ± 0.35bc	−8.58 ± 0.04a	28.52 ± 0.15ab	29.79 ± 0.15a	106.74 ± 0.03a	76.81 ± 0.49a
Buffalo spinach	48.47 ± 0.05ab	−7.22 ± 0.01ab	27.14 ± 0.43b	27.90 ± 0.08b	105.01 ± 0.07a	64.89 ± 1.83bc
Ivy gourd	41.14 ± 0.08c	−5.67 ± 0.06c	21.22 ± 0.06 cd	21.97 ± 0.06d	104.95 ± 0.13ab	57.53 ± 0.16d
White goosefoot	54.36 ± 0.21a	−7.34 ± 0.01ab	30.88 ± 0.05a	31.74 ± 0.05a	103.36 ± 0.02b	67.71 ± 0.57b
Spiny amaranth	49.79 ± 0.65ab	−5.89 ± 0.02b	20.58 ± 0.31d	21.41 ± 0.31d	105.97 ± 0.20a	41.84 ± 1.61e

*Note:* Values are represented as the mean ± standard deviation of the mean. Means followed by different lowercase letters in the same column indicate significant differences at *p* < 0.05. L*‐Lightness; a*‐red/green; b*‐yellow/blue.

This darkening may be attributed to the high green coloration observed in the samples, which was supported by the values of the green (−)/red (+) components (a*), ranging from −5.67 to −8.58 (Table 4). The most intense green coloration was found in *Water spinach*, *Gima*, *Ivy gourd*, and *Spiny amaranth*. The blue (−)/yellow (+) component (b*) was recorded in a range of 20.58–30.88 (Table 4), indicating a lower presence of blue and a slight increase in yellowness in the edible weed powder. This impacted the hue angle, which varied between 21.41° and 31.74°, and the color saturation (chroma), with values ranging from 103.75 to 106.74 (Table 4).

Moreover, the browning index values (ranging from 41.84% to 76.81%) also reflect the browning and degradation of green color in the edible weed powder (Table 4). Among the weed powders, Spiny amaranth retained its green color and exhibited the least browning, followed by Gima and Ivy gourd. In contrast, Alligator weed demonstrated the highest browning index, followed by Water spinach, White goosefoot, and Buffalo spinach.

The process of browning is a complex phenomenon influenced by several dynamics, including substrate levels, enzymatic activity, and the presence of heat‐sensitive elements such as ascorbic acid, proteins, carbohydrates, and different pigment groups like chlorophylls and carotenoids. Additional factors, including heat, light, and air, can also contribute to the browning reaction and tissue injury (Ahmed et al. 2010; Correia et al. 2009; Kamal et al. 2020). The observed reduction in green color among the weed powder samples may be triggered by enzymatic and nonenzymatic reactions that occur during shade drying operations (García‐Martínez et al. 2012).

### 3.4 Evaluation of Bioactive Compounds of Edible Weed Powder

The ability of fruits, vegetables, and their by‐products to provide health benefits is largely dependent on the presence of bioactive phytochemicals such as phenolic acids, carotenoids, and vitamins (Tiburski et al. 2011). Given the potential as a reservoir of nutrients and phytochemicals, the current study investigated the levels of ascorbic acid, total carotenoids, *β‐carotene*, chlorophyll, total phenolics, flavonoids, and antioxidant activity in wild edible weeds.

#### 3.4.1 Ascorbic Acid Content

Ascorbic acid, recognized as one of the most potent antioxidant compounds, is associated with a reduced risk of cancer in humans (Almeida et al. 2011). The concentration of ascorbic acid in edible weed powder showed substantial variability, ranging from 23.53 to 156.66 mg/100 g (Table 5). Among the various samples, White goosefoot exhibited the highest ascorbic acid content, while Ivy gourd demonstrated the lowest. Notably, there was a significant difference (*p* < 0.05) in ascorbic acid levels across the samples.

**TABLE 5:** Bioactive compounds of wild edible weeds powder.

Sample	AAC (mg/100 g)	TCC (mg/100 g)	*β‐carotene* (mg/100 g)	Chlorophyll‐a (mg/100 g)	Chlorophyll‐b (mg/100 g)	Total chlorophyll (mg/100 g)	TPC (mg GAE/100 g)	TFC (mg CE/100 g)
Water spinach	23.53 ± 1.77d	46.24 ± 0.81e	2.18 ± 0.02a	2.59 ± 0.04ab	4.95 ± 0.05a	7.54 ± 0.01a	3046.67 ± 9.93a	1973.72 ± 31.96a
Gima	32.12 ± 1.66c	53.10 ± 0.35d	2.12 ± 0.03a	2.57 ± 00.07ab	4.88 ± 0.01a	7.45 ± 0.06a	685.73 ± 7.39e	154.28 ± 1.46e
Alligator weed	23.53 ± 1.02d	72.08 ± 4.11c	2.18 ± 0.01a	2.63 ± 0.01ab	4.87 ± 0.01a	7.49 ± 0.02a	948.07 ± 10.74c	435.53 ± 12.86d
Buffalo spinach	30.78 ± 2.39c	47.45 ± 0.49e	2.18 ± 0.04a	2.64 ± 0.01ab	4.82 ± 0.01a	7.45 ± 0.01a	1312.97 ± 11.97b	479.22 ± 6.67d
Ivy gourd	21.53 ± 1.73d	83.20 ± 1.04b	2.62 ± 0.01a	2.73 ± 0.02a	4.80 ± 0.03a	7.53 ± 0.04a	920.80 ± 7.45c	516.14 ± 18.83c
White goosefoot	156.66 ± 8.57a	86.77 ± 0.28b	2.10 ± 0.01a	2.56 ± 0.02ab	4.80 ± 0.02a	7.35 ± 0.04a	1498.00 ± 9.26b	679.75 ± 9.34b
Spiny amaranth	45.29 ± 1.77b	139.24 ± 2.77a	2.08 ± 0.03a	2.76 ± 0.03a	4.93 ± 0.08a	7.68 ± 0.05a	813.63 ± 6.27d	514.50 ± 27.02c

*Note:* Values are represented as the mean ± standard deviation of the mean. Means followed by different lowercase letters in the same column indicate significant differences at *p* < 0.05.

Abbreviations: AAC, ascorbic acid content; TCC, total carotenoid content; TFC, total flavonoid content; TPC, total phenolic content.

One of the primary reasons for this variation can be attributed to the specific weed species included in this study. Additionally, several studies have established that ascorbic acid is highly sensitive to heat (Raja et al. 2019). Exposure to factors such as oxygen, pH, and other environmental parameters has been shown to significantly contribute to the degradation of ascorbic acid (Wang et al. 2018). Consequently, the prolonged exposure of edible weed plants to shade‐drying conditions may be a critical factor leading to the observed loss of ascorbic acid.

#### 3.4.2 Evaluation of Pigment Concentration

Pigments such as carotenoids, chlorophyll, and *β‐carotene* play a vital role in regulating metabolic activities, primarily by reducing the risks of heart disease and cancer due to their potent antioxidant activity and beneficial health effects (Crupi et al. 2023; Lu et al. 2021). Table 5 presents the total carotenoid concentration in the edible weed powder, which varied significantly among the samples (*p* < 0.05), ranging from 46.24 to 139.24 mg/100 g. The highest levels of total carotenoids were observed in sample Spiny amaranth, while the lowest were found in Water spinach powder.

In contrast, the *β‐carotene* content (Table 5) did not exhibit significant variation, with values ranging between 2.08 and 2.62 mg/100 g. Similar findings were noted for chlorophyll‐a, which ranged from 2.57 to 2.76 mg/100 g, and chlorophyll‐b, fluctuating from 4.80 to 4.95 mg/100 g. The total chlorophyll content varied from 7.35 to 7.68 mg/100 g.

The findings indicate that extended drying times, as experienced in this study, can significantly impact the stability of pigments in food materials. Additionally, factors such as acids, light exposure, and oxidative reactions—especially in the presence of metallic substances—can further trigger pigment degradation in the edible weed powders (Cao et al. 2024; Xu et al. 2021).

#### 3.4.3 Total Phenolic Content

Polyphenols are recognized as one of the primary classes of bioactive substances found in food, playing a crucial role in biological functions that combat chronic diseases such as cancer, cardiovascular disorders, and inflammation. As shown in Table 4, the total polyphenol content (TPC) of the edible weed powder varied significantly, ranging from 685.73 to 3046.67 mg GAE/100 g. The results indicate a substantial variation (*p* < 0.05) in TPC among the different weed powders. The highest concentration of phenolic compounds was observed in Water spinach, followed by White goosefoot and Buffalo spinach, while the lowest concentration was found in Gima.

The release of polyphenolic substances is often linked to the maturity and chemical structure of the weed plant, as these compounds are typically bound to the plant matrix (Tan et al. 2020). Additionally, the activity of enzymes such as polyphenol oxidase contributes significantly to the release of phenolic compounds (Paul and Das 2018). Moreover, the conjugation of polyphenols with other constituents of the dietary matrix—including proteins, carotenoids, sugars, and organic acids—as well as geographical variations or extraction methods may further influence the amount of phenolics present (Aryal et al. 2019; Burri et al. 2017).

#### 3.4.4 Total Flavonoid Content

Flavonoids are important classes of phytochemicals present in plant‐based food matrices that enhance antioxidant activity. Table 5 indicates that the total flavonoid concentration in the edible weed powders varied significantly (*p* < 0.05) among the samples, ranging from 154.28 to 1973.72 mg CE/100 g of dry sample. Notably, sample Water spinach retained a significantly higher amount of flavonoids compared to the other samples.

According to the literature, factors such as genetic diversity and biological, environmental, seasonal, and year‐to‐year variations can have a substantial impact on the flavonoid content in vegetables (Aryal et al. 2019; Kumar and Roy 2018). Regular consumption of these weedy, leafy vegetables can contribute considerable amounts of flavonoids to local diets. High intakes of flavonoids have been associated with various health benefits, including protection against cardiovascular diseases, stroke, and cancer (Obeng et al. 2020).

### 3.5 Evaluation of Antioxidant Activity

**FIGURE 1:**
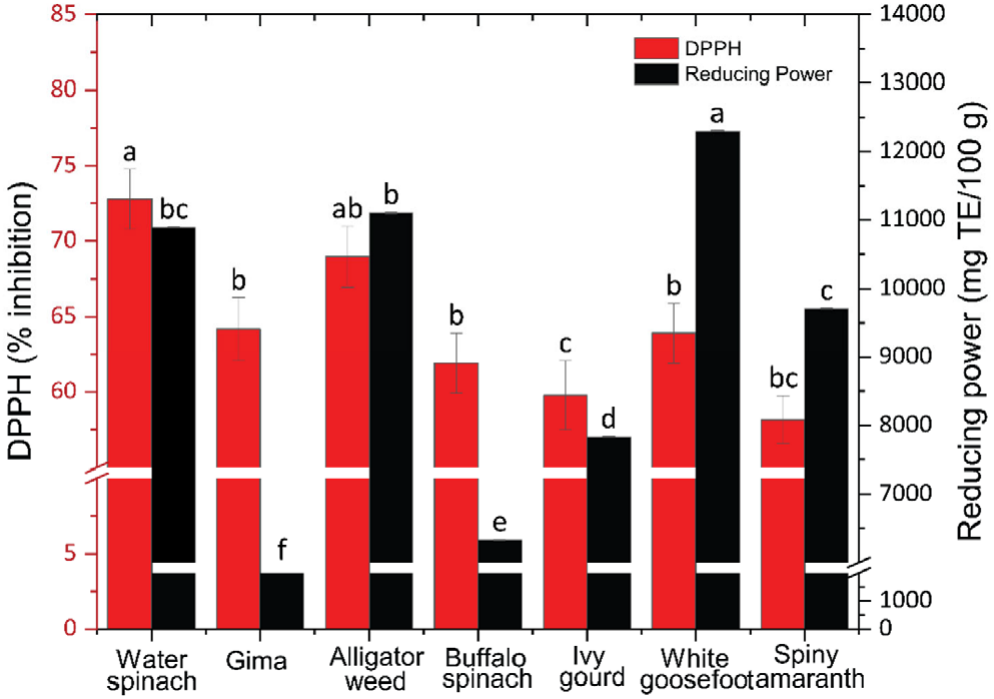
DPPH free radical scavenging activity and reducing power of the analyzed edible weeds.

The DPPH (2,2‐diphenyl‐1‐picrylhydrazyl) assay is a well‐established method for measuring the free radical scavenging activity of plant extracts, providing insights into their antioxidant capacity (Baliyan et al. 2022). In this study, the antioxidant properties of seven edible weeds were assessed through the DPPH radical scavenging activity and reducing power assays, and the results illustrated in Figure 1.

As shown in Figure 1, the DPPH‐RSA values ranged from 58.15% to 72.77% inhibition across the various weed extracts, with the highest inhibition observed in sample Water spinach, followed closely by Alligator weed, Gima, White goosefoot, and others. Notably, all weed extracts demonstrated over 50% free radical scavenging activity, indicating their effectiveness.

In addition, the reducing power assay results ranged from 6328.00 to 12298.23 mg TE/100 g, with sample White goosefoot exhibiting the highest reducing power, followed by Alligator weed and Water spinach (Figure 1). Overall, it is evident that all samples exhibited potent antioxidant activity.

Previous studies have established that the antioxidant capacity of food matrices is closely linked to their electron transfer ability, thus serving as a significant indicator of antioxidant activity (Ajila et al. 2007). The antioxidant capacity observed in the studied samples may be attributed to their higher content of phenolics and flavonoids, along with carotenoids (Ayele et al. 2022; Chandrasekara and Josheph Kumar 2016).

### 3.6 Multivariate Analysis

**FIGURE 2:**
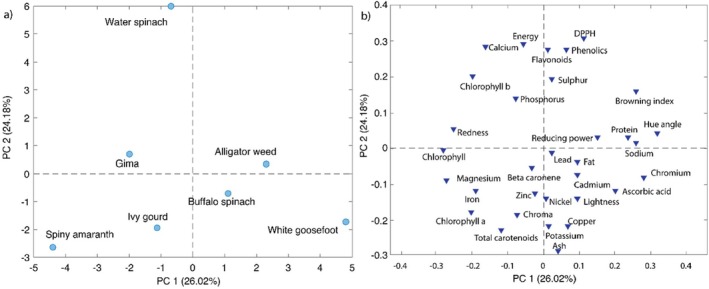
Principal component analysis (a) score plot, (b) loading plot.

To reduce the dimensionality of the data, principal component analysis (PCA) was carried out. The PCA model was built on auto scaled data. Four principal components were chosen, which explained 82% of the total variance. In Figure 2, the first two components were presented since they explained more than 50% of the total variance. From the score plot, it can be seen that ivy gourd and spiny amaranth appeared similar. Sample weeds that lie in the same coordinate appeared similar. It can be seen that water spinach is different from the other samples (Figure 2a).

From the loading plot it is evident that water spinach is mostly attributed to total phenolics, flavonoids, DPPH and energy content. It is negatively correlated with minerals such as zinc, nickel, copper, potassium. Spiny amaranth and ivy gourd is positively correlated with iron content and chlorophyll a and total carotenoids. White goosefoot is negatively correlated with chlorophyll b (Figure 2b).

**FIGURE 3:**
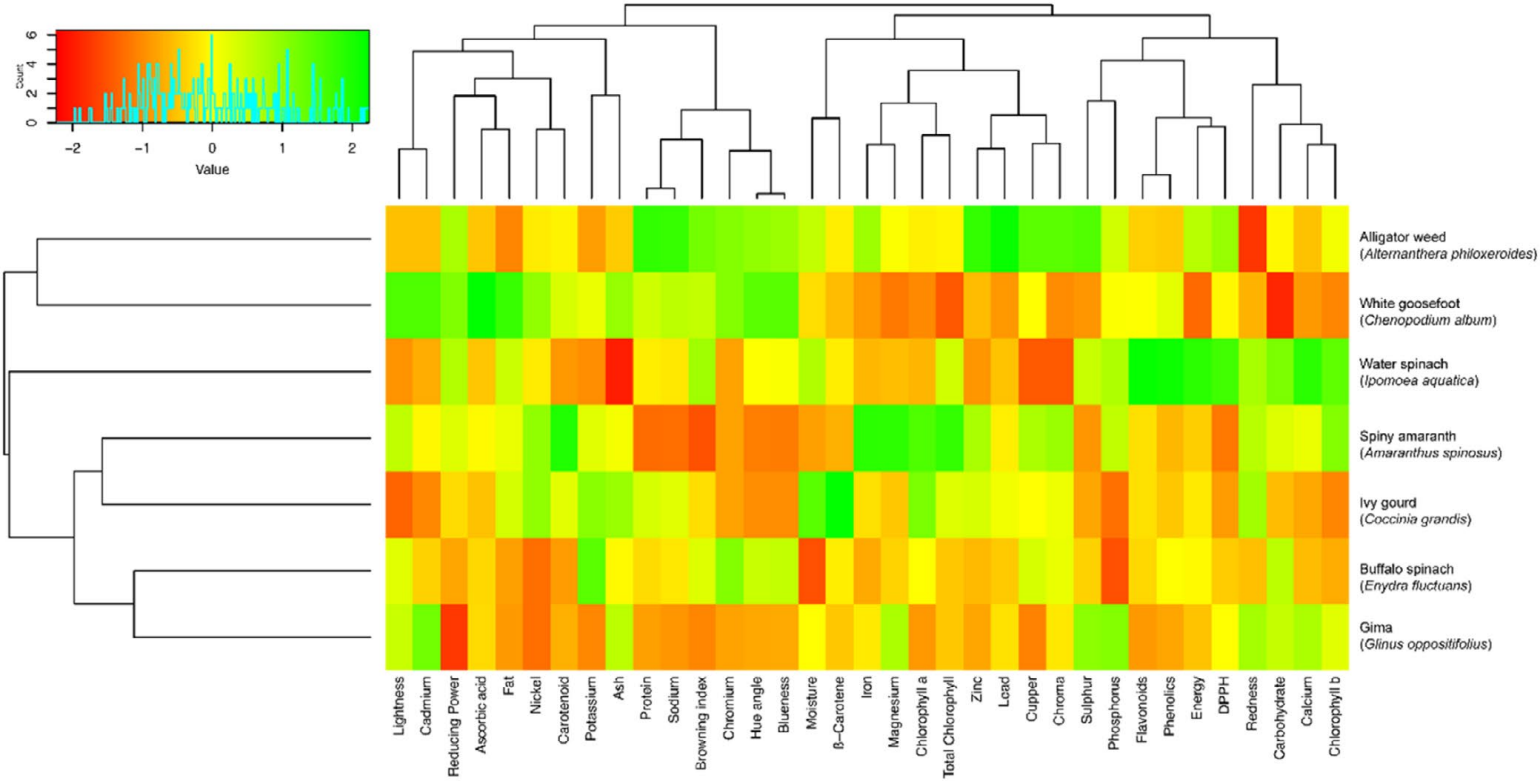
Clustering of the studied weed samples.

The results obtained by the cluster analysis are shown in the heatmap and dendrogram (Figure 3). Cluster analysis revealed the existence of three clusters. The first cluster consists of buffalo spinach and gima, while the second and third clusters are formed of spiny amaranth & ivy guard and alligator weed & goosefoot, respectively. Furthermore, the first two clusters were connected to water spinach and finally were connected to the cluster of alligator weeds (Figure 3). PCA and cluster analysis results showed similar patterns that the weed composition had a higher influence on weed differentiation, which is supported by the univariate results presented in Tables 2, 3, 4, 5 and Figure 1. These findings were consistent with the data reported in the literature (Eddoud et al. 2018; Harrington et al. 2006; Suwignyo et al. 2017).

The scope of the sampling in this study was restricted to a specific geographic area, which might not encompass the full environmental variability affecting trace element accumulation. Moreover, analytical limitations include the use of standard conventional methods for nutritional profiling, which may lack the specificity of advanced techniques such as chromatography. Furthermore, assessing only raw dried powder without considering cooking or further processing in this study may affect the understanding of real‐world nutrient intake and toxin reduction.

## 4 Conclusions

This study provides a comprehensive analysis of seven commonly consumed Bangladeshi edible weeds, highlighting their substantial nutritional and bioactive compound content alongside potential safety concerns. Notably, the weed powders exhibited high levels of protein, ash, and antioxidant constituents such as ascorbic acid, carotenoids, phenolics, and flavonoids, indicating their promise as functional foods with health‐promoting properties.

However, significant variability was observed in trace metal concentrations, with some species accumulating higher levels that could pose health risks if consumed regularly over time. These findings show the importance of regular monitoring of heavy metals in edible weeds to ensure consumer safety.

Considering their substantial nutritional and bioactive profiles, these weed species can be regarded as valuable alternative sources of functional food, particularly in regions facing dietary limitations or seeking sustainable, locally available food options. Future research should focus on employing advanced analytical techniques to achieve a more detailed profile of individual bioactive compounds and establishing safe consumption thresholds as well as exploring processing methods that can ensure bioavailability of key phytochemicals and reduce toxic element levels. Overall, this work supports the potential utilization of edible weeds in functional foods and calls for policies incorporating safety assessments to promote their sustainable and safe use.

## Author Contributions

**Md. Mostafa Kamal:** conceptualization (equal), formal analysis (equal), funding acquisition (equal), investigation (equal), methodology (equal), project administration (equal), resources (equal), supervision (equal), writing – original draft (equal), writing – review and editing (equal). **Somiya Haque:** data curation (equal), formal analysis (equal), methodology (equal), writing – original draft (equal). **Tanim Kazi Suvo:** data curation (equal), formal analysis (equal), methodology (equal), writing – original draft (equal). **Prity Akter:** data curation (equal), formal analysis (equal), methodology (equal), writing – original draft (equal). **Md. Suman Mia:** data curation (equal), formal analysis (equal), writing – original draft (equal). **Md. S. M. Sifat Shah:** data curation (equal), formal analysis (equal), visualization (equal). **Md. Nahidul Islam:** investigation (lead), supervision (lead), validation (lead), visualization (lead), writing – original draft (equal), writing – review and editing (equal). **Md. Golam Ferdous Chowdhury:** methodology (equal), resources (equal), supervision (equal).

## Conflicts of Interest

The authors declare no conflicts of interest.

## Data Availability

The data that support the findings of this study are available on request from the corresponding author.
